# Supplementary material for: Evaluation of the Reporting Standard Guidelines of Network Meta-Analyses in Physical Therapy: A Systematic Review
Source: Healthcare (Basel). 2022 Nov 25;10(12):2371. doi: 10.3390/healthcare10122371 (PMC9778181; doi:10.3390/healthcare10122371)
Supplement: Supplementary file 1 [file healthcare-10-02371-s001.zip › healthcare-2000199-supplementary.pdf]

Table S1. Summary of the included studies

No	Author (country*)	Year	Journal	SR/MA	The number and design of included studies	Population/patients/defects	Intervention	Comparison between interventions	Outcome
1	Scapini et al (Brazil)	2019	Journal of Physiotherapy	SR & MA	33 randomized trials involving 1254 participants	Adults requiring hemodialysis for end-stage renal disease	Aerobic, resistance and combined exercise training	Control group (no exercise or placebo) and to each other	Aerobic capacity and arterial blood pressure at rest, and hemodialysis efficiency
2	Li et al (China)	2018	Medicine	SR & MA	19 randomized trials with 1676 patients	Adults diagnosed with plantar fasciitis	Extracorporeal shock wave, ultrasound, low-level laser therapy, noninvasive interactive neurostimulation, and pulsed radiofrequency treatment	Each other or with a sham group	Pain relief (visual analog scale, the numerical rating scale pain score, the pain subscale of the validated foot function index)
3	Chiang et al (Taiwan)	2019	Archives of Physical Medicine and Rehabilitation	SR & MA	19 randomized trials with 753 patients	acute and subacute patients with post-stroke dysphagia within 90 days	Noninvasive neurostimulation therapies (including rTMS, tDCS, sNMES, pulsed electrical stimulation [PES])	placebo group, sham stimulation and no stimulation	Swallowing function evaluated before and after neurostimulation therapy
4	Lai et al (Taiwan)	2019	Physical Therapy in Sport	SR & MA	6 randomized trials with 263 participants	adults (age range: 18-50 years)	PNF stretching exercise Kinesiotaping	usual care, kinesiotaping	Length of the pectoralis minor
5	Lai et al (Taiwan)	2018	Age and Ageing	SR & MA	30 trials involving 1405 participants	Adults aged 60 and over (age range: 60-92 years)	Resistance training, endurance training and whole-body vibration	usual care control group, trials incorporating a placebo-based intervention	lean body mass, muscle strength and physical performance

6	Wang et al (China)	2019	Aging Clinical and Experimental Research	MA	18 RCTs with 1364 participants	Adults aged 50 years or older with mild cognitive impairment	Different types of exercises (aerobic exercise, resistance exercise, mind-body exercise, exergame)	Control group (stretching, education, usual lifestyle, and social recreational activities)	Global cognitive
7	Tsikopoulos et al (Greece)	2018	Journal of Science and Medicine in Sport	SR & MA	19 RCTs with 622 participants	Patients with lateral mechanical ankle instability and/or recurrent ankle sprains and/or lateral functional ankle instability	Non-surgical treatments (Balance training, Strengthening exercise, Vibration training, Sensory-targeted ankle rehabilitation strategies)	Control group	Dynamic postural control of the lower extremity through measurable reach distances: star-excursion balance test
8	Zeng et al (China)	2015	Osteoarthritis and Cartilage	SR & MA	27 RCTs with 1249 participants	Patients with knee osteoarthritis	Six kinds of electrical stimulation therapies; high-frequency transcutaneous electrical nerve stimulation, low-frequency transcutaneous electrical nerve stimulation, interferential current, PES, and noninvasive interactive neurostimulation	Control interventions (sham or blank)	Pain relief
9	Uthman et al (UK)	2013	BMJ	MA	60 RCTs with 8218 patients	Adults with an established clinical or radiographic diagnosis of knee or hip osteoarthritis	Flexibility, Strengthening, Aerobic (land- or water-based)	other forms of exercise or no exercise control group	In agreement with international consensus regarding the core set of outcome

						according to accepted criteria	therapeutic exercise intervention		measures for clinical trials in osteoarthritis, the trials needed to include assessment of at least one of self-reported pain and function
10	Pan et al (China)	2018	International Journal of Behavioral Nutrition and Physical Activity	SR & MA	37 RCT studies with 2208 patients	Patients with type 2 diabetes mellitus (T2DM)	Different exercise training modalities (supervised aerobic exercise, unsupervised aerobic exercise, anaerobic exercise, supervised resistance exercise, unsupervised resistance exercise, combined exercise, flexibility exercise and no exercise)	Control group	Glycemic control, cardiovascular risk factor, and weight loss
11	Schwingshackl et al (Australia)	2014	Diabetologia	SR & MA	14 RCT studies with 915 patients	Patients with T2DM	Aerobic exercise training (AET), resistance training (RT), combined aerobic and resistance training (CT)	AET vs. RT and/or CT vs. AET and/or CT vs. RT	Glycemic control and blood lipids
12	Schwingshackl et al (Australia)	2013	PLOS ONE	SR & MA	15 RCT studies with 741 patients	Overweight and obese individuals	AET, RT, CT	AET vs. RT and/or CT vs. AET and/or CT vs. RT	Anthropometric outcomes, blood lipids, and cardiorespiratory fitness parameters

13	Cheung et al (Hong Kong)	2020	Physiotherapy	SR & MA	6 RCT studies with 418 patients	Patients diagnosed with carpal tunnel syndrome (CTS) by electrodiagnostic test (including nerve conduction studies), electromyography, imaging or any other clinical criteria	Low-level laser therapy, sham laser, ultrasound	Other conservative treatments (splinting)	1) Changes in CTS-related pain, measured by validated scale 2) Changes in CTS symptom severity, measured by the Boston Carpal Tunnel Syndrome Symptom Severity Scale 3) Changes in CTS functional status, measured by the Boston Carpal Tunnel Syndrome Function Severity Scale
14	Zhang et al (China)	2019	American Journal of Physical Medicine & Rehabilitation	MA	32 RCT studies with 3228 patients	Older adults who experience osteoarthritis, irrespective of sex and joint location	Different nonpharmacological interventions (including walking, strengthening exercise, Yoga, aquatic exercise, healing touch, and Tai Chi)	Control group	Pain intensity
15	Goh et al (UK)	2019	Sports Medicine	SR & MA	103 RCT studies with 9134 patients	Participants with knee OA, hip OA, or mixed knee and hip OA diagnosed clinically and/or radiographically	Different exercise programs (aerobic, mind-body, strengthening, flexibility/skill, or mixed) without additional active treatment (e.g.,	Usual care/waiting list or a different exercise as the control group	improving pain, function, objective performance and quality of life for knee and hip OA

							analgesics) as the intervention		
16	Tang et al (China)	2019	Journal of Clinical Neuroscience	MA	19 RCT studies with 920 patients	Participants with Parkinson disease	Exercise interventions such as dance, Qigong, tango, RT, Taichi and yoga	Control group	Six-minute walk, gait velocity, UPDRS III, PD questionnaire-39, timed up and go and Berg balance test
17	Owen et al (Australia)	2019	Br J Sports Med	MA	89 randomized controlled/clinical trials	Adults with non-specific chronic low back pain	Specific modes of exercise training (Resistance, Stabilization/motor control, Pilates, Yoga, McKenzie, Flexion, Aerobic, Water-based, Stretching, Other, Multimodal)	Control (True, Therapist hands-on, Therapist hands-off)	Subjective pain intensity, subjective physical function, objective trunk muscle strength, objective trunk muscle endurance, subjective analgesic pharmacotherapy use or subjective mental health
18	Hilfiker et al (Switzerland)	2018	Br J Sports Med	SR & MA	245 randomized or quasi-randomized trials	Patients during and after cancer treatment	all kinds of exercise or other non-pharmaceutical interventions such as cognitive–behavioral or relaxation interventions on cancer-related fatigue (CRF) or vitality in	Control group	CRF

							patients with CRF during or after active cancer treatments		
19	Wu et al (Taiwan)	2017	Archives of Physical Medicine and Rehabilitation	SR & MA	14 RCT studies with 1105 patients	Participants with chronic calcific tendinitis of the shoulder	Various nonoperative treatments (UGN, H-FSW, RSW, L-FSW, ultrasound therapy, and TENS)	Control group such as sham treatment or physiotherapy alone	Pain severity, functional status of shoulder in patients with calcific tendinitis, and the ratio of complete resolution of calcific deposition
